# Effects of SOX10 on the proliferative, invasive, migratory, and epithelial–mesenchymal transition abilities of triple-negative breast cancer cells

**DOI:** 10.3389/fonc.2026.1769833

**Published:** 2026-05-18

**Authors:** Shuai Wang, Linfang Jin

**Affiliations:** Wuxi Ninth People’s Hospital Affiliated to Soochow University/Wuxi Orthopedic Institute, Wuxi, Jiangsu, China

**Keywords:** cellular biological behavior, epithelial-mesenchymal transition, SOX10, triple negative breast cancer, Wnt/β-catenin pathway

## Abstract

**Introduction:**

Triple-negative breast cancer (TNBC) is a highly aggressive subtype of breast cancer with limited targeted therapeutic options. Sry-related HMG-box gene 10 (SOX10) has been implicated in tumor progression in various malignancies, but its functional role and underlying mechanism in TNBC remain unclear. This study aimed to investigate the effect of SOX10 on TNBC cell proliferation, metastasis, and epithelial–mesenchymal transition (EMT), and to explore its association with the Wnt/β-catenin signaling pathway.

**Methods:**

HCC1937 cells (high endogenous SOX10 expression) and MDA-MB-453 cells (low endogenous SOX10 expression) were used for SOX10 knockdown and overexpression experiments. Wnt pathway activators and inhibitors were applied in rescue experiments to validate the mechanistic link. Cell proliferation was assessed by CCK-8 assay; migration and invasion were evaluated by scratch wound and Transwell assays, respectively. The expression levels of EMT markers and Wnt/β-catenin pathway-related proteins were quantified by Western blot analysis.

**Results:**

SOX10 overexpression significantly promoted proliferation, migration, and invasion in both TNBC cell lines, while SOX10 knockdown markedly inhibited these abilities (all P<0.05). SOX10 upregulated the expression of mesenchymal markers (Vimentin, N-cadherin) and Wnt/β-catenin pathway components (WNT1, nuclear β-catenin, C-myc, Cyclin D1), and downregulated the epithelial marker E-cadherin (all P<0.05). Rescue experiments confirmed that Wnt pathway activation reversed the EMT suppression induced by SOX10 knockdown, while Wnt pathway inhibition attenuated the EMT promotion caused by SOX10 overexpression (all P<0.05).

**Discussion:**

SOX10 promotes malignant biological behaviors in TNBC cells by activating the Wnt/β-catenin signaling pathway and inducing EMT. These findings suggest that the SOX10-Wnt/β-catenin axis may serve as a potential therapeutic target for TNBC, warranting further mechanistic and translational investigation.

## 1 Introduction

According to the 2020 data on breast cancer (BC) released by the International Agency for Research on Cancer (IARC), it remains one of the major malignant tumors endangering women’s health worldwide (1). Its incidence ranks first globally and fourth in China. Based on its histological morphology, triple-negative breast cancer (TNBC), a highly heterogeneous category of BC, can be further subdivided into basal-like BC and metaplastic BC. Clinically, TNBC is characterized by poor differentiation, high invasiveness, early recurrence and distant metastasis, short survival time, and poor prognosis (2). Due to the lack of ER, PR, and Her-2 expression in TNBC, coupled with the absence of specific target genes, highly effective endocrine and targeted therapies remain unavailable. Therefore, chemotherapy currently remains the sole comprehensive treatment method for TNBC. However, treating this disease remains a challenging and urgent problem in clinical practice (3).

Sry-box transcription factor 10 (SOX10), a member of the Sox gene family, contributes to the biological activities of melanocytes and Schwann cells in peripheral nerves (4). Additionally, SOX10 immunohistochemical labeling is used in the diagnosis of melanoma, salivary gland myoepithelioma, and tumors originating from peripheral nerve sheath and sweat gland ducts in the skin (5). Recently, SOX10 has been confirmed to induce the occurrence and progression of many tumors other than breast cancer (BC), primarily by modulating the proliferation, metastasis, and other biological functions of tumor cells (6).

In tumors, the epithelial-mesenchymal transition (EMT) plays a critical role in infiltration and metastasis (7). The hallmark proteins of EMT include epithelial markers such as E-cadherin and β-catenin, as well as stromal markers like vimentin, N-cadherin, and matrix metalloproteinases (MMP2, MMP3, and MMP9). Following EMT, cells exhibit downregulation of epithelial markers and upregulation of stromal phenotype markers (8). The occurrence of EMT is a highly complex process, involving multiple signaling pathways and various molecular mechanisms (15, 16). Currently, many studies have confirmed that the Wnt signaling pathway is crucial for EMT development in tumors (9, 10), with most tumor cells primarily utilizing β-catenin to mediate the EMT process through the classical Wnt/β-catenin signaling pathway (11). A previous study found that SOX10 in nasopharyngeal carcinoma also promotes tumor growth and metastasis through EMT induction, but the specific pathway involved has not been further investigated (6). Another study found that in hepatocellular carcinoma, directly activating the Wnt/β-catenin pathway promotes cell proliferation, invasion, and metastasis, leading to increased malignant potential (12). Our previous research (26) indicated that SOX10 protein expression may be associated with a poor prognosis in TNBC, and SOX10 may promote EMT in TNBC cells. However, the molecular mechanism is still unclear. Here, we have further investigated the precise action of SOX10 on the biological behavior of TNBC cells and determined the EMT-related and Wnt/β-catenin pathway-related factors at the cytological level.

## 2 Materials and methods

### 2.1 TNBC cell lines

Four types of TNBC cell lines (HCC1937, MDA-MB-231, MDA-MB-468, and MDA-MB-453) were used in this study and purchased from ATCC (United States). All cell lines were authenticated by short tandem repeat (STR) profiling upon receipt and periodically during the study to confirm cell line identity and exclude cross-contamination. Mycoplasma contamination was routinely tested using a PCR-based mycoplasma detection kit (e.g., MycoAlert Mycoplasma Detection Kit, Lonza, Basel, Switzerland) every 2 weeks throughout the experimental period, and all cell lines were confirmed to be mycoplasma-free prior to and during the experiments. HCC1937 was maintained in RPMI1640 medium, which was supplemented with fetal bovine serum (FBS, 10%, Gibco, Waltham, Massachusetts, United States) and 1% penicillin–streptomycin (Gibco, Waltham, Massachusetts, United States), and incubated at 37 °C in a 5% CO_2_ incubator. The other three cell lines were maintained in L15 medium supplemented with 10% FBS and 1% penicillin–streptomycin, and cultured at 37 °C under normal atmospheric conditions. TNBC cells were seeded at 3 × 10^5^ cells/well in 6-well plates during the logarithmic growth phase. SOX10 expression levels were first screened across all four cell lines by qRT-PCR and Western blot analysis. Based on the screening results, HCC1937 (with the highest SOX10 expression) and MDA-MB-453 (with the lowest SOX10 expression) were selected for subsequent functional experiments to investigate both knockdown and overexpression effects of SOX10.

### 2.2 Cell transfection

Four types of vectors were used in this study: (1) siRNA negative control (siNC), (2) siRNA targeting SOX10 (siSOX10) for gene knockdown, (3) overexpression negative control vector (NC), and (4) SOX10 overexpression vector (SOX10-OE). All vectors were synthesized by Jiangsu Kaiji Biotechnology Co., Ltd, Jiangsu Province, China. Both HCC1937 and MDA-MB-453 cells were transfected with siNC/siSOX10 for knockdown experiments and NC/SOX10-OE for overexpression experiments, respectively. The siRNA sequences targeting human SOX10 were as follows: siSOX10–1 sense 5’-GAACGAAAGUGACAAGCGC-3’, antisense 5’-GCGCUUGUCACUUUCGUUC-3’; siSOX10–2 sense 5’-GAGAUCAGCCACGAGGUAA-3’, antisense 5’-UUACCUCGUGGCUGAUCUC-3’. Lipofectamine 2000 (Invitrogen, Carlsbad, California, United States) was used for transfection according to the manufacturer’s instructions. Transfection efficiency was verified by qRT-PCR and Western blot analysis at 48 h post-transfection. Both siRNA sequences produced consistent knockdown of SOX10 at both the mRNA and protein levels as confirmed by qRT-PCR and Western blot. siSOX10–1 was selected for all subsequent functional experiments based on its higher knockdown efficiency, while siSOX10–2 was used in parallel as an independent confirmatory sequence to exclude off-target effects. Unless otherwise stated, “siSOX10” in the figures and results refers to siSOX10-1.

### 2.3 CCK-8 measurement

HCC1937 and MDA-MB-453 cells (5×10^3^ cells/well) were separately seeded into a 96-well plate. After transfection, 15 μL/well of CCK8 reagent was added to the cells, which had been cultured for 24, 48, and 72 h, respectively. The plates were then incubated at 37 °C in a 5% CO_2_ incubator for 3 h. The absorbance at 450 nm (OD_450nm_) was measured using a Multiskan FC microplate reader (Thermo Scientific, USA). For each experimental group, six technical replicate wells were set up at each time point (24, 48, and 72 h), and the entire experiment was independently repeated three times (n = 3 biological replicates). The mean OD450 values from the six technical replicates within each biological replicate were used for statistical analysis. Data are expressed as mean ± SD across the 3 independent experiments. The cell proliferation rate was calculated with the following formula: Proliferation rate (%) = (OD value of experimental group − OD value of blank well)/(OD value of control group − OD value of blank well) × 100%.

### 2.4 Detection of cell migration by scratch assay

HCC1937 and MDA-MB-453 cells in the logarithmic growth phase were seeded into 6-cm dishes. When confluent to 80%, the cells were treated according to the protocols for different groups. Cell scratches were made using a 200-µL gun head, held perpendicular to the dish, ensuring consistent scratch width. After discarding the cell culture medium, cells were washed three times with PBS. Images of the same marked areas for each group of cells were collected under the microscope at 0 and 24 h, respectively. For each scratch, images were acquired at the exact same marked positions at 0 h and 24 h using an IX73 inverted phase-contrast microscope (Olympus, Japan) at 100× magnification, with three non-overlapping random fields selected per well. The scratch width at each time point was measured by ImageJ 1.54v software (NIH, Bethesda, MD, USA), with three parallel measurements performed per field. The cell migration rate was calculated using this formula: Migration rate (%) = (scratch width at 0 h - scratch width at 24 h)/scratch width at 0 h × 100%. The experiment was independently repeated three times. To minimize the potential contribution of cell proliferation to wound closure, all scratch assay experiments were performed within a 24-h window, during which the proliferation rate is unlikely to substantially confound migration measurements. Furthermore, consistent results obtained from the Transwell migration assay, which is independent of proliferative activity, corroborate the SOX10-mediated regulation of cell migration.

### 2.5 Detection of cell invasion by transwell assay

Matrigel (BD Biosciences, Franklin Lakes, New Jersey, United States, Cat. No. 356234; protein concentration: ~8–10 mg/mL), stored at -20°C, was thawed on ice overnight and kept at 2 °C –8°C. One hundred microliters of Matrigel was diluted with 300 μL of pre-cooled serum-free culture medium (1:3 dilution) to achieve a final working concentration of approximately 2–2.5 mg/mL, and mixed thoroughly on ice. This Matrigel concentration was kept consistent across all experimental groups. Forty microliters of the culture medium containing Matrigel was added to the upper chamber. The chambers were then maintained at 37°C for 30 min to polymerize the Matrigel into a gel. Cells were prepared as a single-cell suspension using serum-free medium, at a concentration of approximately 5×10^5^ cells/mL. Cell suspension [100 μL (5×10^4^)] and 200-μL culture medium were positioned into the upper level, and cells were then stimulated according to their respective experimental groups. Chemotactic factor was supplied to the lower chamber of the Transwell. The chambers were then incubated for approximately 48 h at 37°C in a 5% CO_2_ incubator. A wet cotton swab was used to gently wipe the Matrigel and the cells from the surface. The upper chamber was carefully removed and labeled with a thread. The chamber was incubated in pre-cooled methanol for 30 min, stained with crystal violet dye for 10 min, then washed with water. The non-migrating cells on the upper layer were gently wiped off with a cotton swab. After fixation and staining, images were acquired using an IX73 inverted microscope (Olympus, Japan) at 200× magnification. For each Transwell chamber, five non-overlapping random fields were selected for cell counting, and the total number of invasive cells per field was quantified manually by two independent investigators in a blinded manner. The average number of invasive cells per field was calculated for each sample, and the experiment was independently repeated three times.

### 2.6 qRT-PCR

HCC1937 and MDA-MB-453 cells were cultured in 6-cm dishes until 50% confluency, then treated according to the protocols of their respective experimental groups. After 24 h, total RNA was extracted using TRIzol reagent, and cDNA synthesis was performed with the PrimeScript RT kit. Primer sequences were referenced from the NCBI database. The primer sequences were as follows: SOX10-Forward 5’-CAGACGCACATCAAGACCCT-3’, SOX10-Reverse 5’-TGCTGGTCGTTGAGGAAGTC-3’ (product length 152 bp); WNT1-Forward 5’-CGATGGTGGGGTATTGTGAAC-3’, WNT1-Reverse 5’-CCGGATTTTGGCGTATCAGAC-3’; β-catenin (CTNNB1)-Forward 5’-AAAGCGGCTGTTAGTCACTGG-3’, β-catenin-Reverse 5’-CGAGTCATTGCATACTGTCCAT-3’; GAPDH-Forward 5’-GGAGCGAGATCCCTCCAAAAT-3’, GAPDH-Reverse 5’-GGCTGTTGTCATACTTCTCATGG-3’ (internal reference). Amplification was performed using the SYBR Premix Ex Taq II kit on an ABI 7500 system. Amplification specificity was confirmed for each reaction through melting curve analysis, with no primer dimers or non-specific amplification products detected. All qRT-PCR reactions were performed with three technical replicates per sample, and at least three independent biological replicates were included for each experimental group. The threshold cycle (Ct) value of each target gene was normalized to the internal reference gene GAPDH, and the relative mRNA expression level was calculated using the 2-^ΔΔCT^ method. The experiment was independently repeated three times.

### 2.7 Western blot analysis

Total proteins were extracted from HCC1937 and MDA-MB-453 cells using RIPA lysis buffer, and protein concentration was quantified using a Thermo Fisher, Waltham, Massachusetts, United States BCA assay. Twenty micrograms of protein per lane was resolved by 10% SDS-PAGE, transferred to PVDF membranes, and blocked with 5% non-fat milk for 1 h at room temperature. The membranes were then incubated overnight at 4°C with primary antibodies from Abcam, Cambridge, United Kingdom targeting: C-myc (ab32072, 1:1000), CyclinD1 (ab134175, 1:2000), E-cadherin (ab40772, 1:500), N-cadherin (ab76011, 1:1000), Vimentin (ab92547, 1:2000), WNT1 (ab15251, 1:500), phospho-GSK-3β (Ser9) (ab75814, 1:1000), SOX10 (ab227680, 1:1000), β-catenin (ab32572, 1:5000), and GAPDH (ab8245, 1:10,000) as a loading control. Following incubation with primary antibodies, membranes were incubated with HRP-conjugated secondary antibodies (1:5000) for 1 h at room temperature. Protein bands were visualized using a ChemiDoc XRS+ chemiluminescence imaging system (Bio-Rad, USA), with exposure time optimized to avoid signal saturation. The gray value of each target protein band was quantified by ImageJ 1.54v software and normalized to the gray value of the internal reference protein GAPDH (for total protein) or Lamin B1 (for nuclear protein, in the case of nuclear β-catenin) from the same sample. For each protein, the relative expression level was calculated by setting the average value of the control group to 1. All Western blot experiments were independently repeated at least three times with consistent results, and representative blots are shown in the figures.

### 2.8 The activation and inhibition treatments of Wnt/β-catenin pathway

The Wnt/β-catenin pathway was activated using CHIR-99021 (a selective GSK-3β inhibitor; MedChemExpress, Monmouth Junction, New Jersey, United States, Cat. No. HY-10182) at a concentration of 5 μM (8), and inhibited using XAV939 (a tankyrase 1/2 inhibitor; MedChemExpress, Cat. No. HY-15147) at a concentration of 10 μM (13). Both compounds were dissolved in dimethyl sulfoxide (DMSO) to prepare 10-mM stock solutions and diluted in complete culture medium prior to use. Compounds were added to the culture medium at 24-h post-transfection, with a treatment duration of 48 h. The final DMSO concentration did not exceed 0.1% (v/v) in all treatment groups, and equal volumes of DMSO were added to control groups. MDA-MB-453 and HCC1937 cells were divided into the following experimental groups: siNC, siSOX10, siNC+Wnt activator, siSOX10+Wnt activator, overexpression NC, overexpression SOX10, overexpression NC+Wnt inhibitor, and overexpression SOX10+Wnt inhibitor. Wnt activators were added to siNC, siSOX10-MDA-MB-453 cells, and siSOX10-HCC1937 cells. Wnt inhibitors were added to overexpressed SOX10-NC, overexpressed SOX10-MDA-MB-453 cells, and overexpressed SOX10-HCC1937 cells.

### 2.9 Statistical analysis

All experiments were performed with a minimum of n=3 independent biological replicates, unless otherwise stated. Each biological replicate was conducted on a separate occasion using independently prepared cell cultures and reagents. All experimental data were analyzed using GraphPad Prism 9.0 software (GraphPad Software, USA). For comparisons among three or more groups, one-way or two-way ANOVA was used, followed by Tukey’s *post-hoc* test for multiple pairwise comparisons. Comparisons between two independent groups were performed using an unpaired two-tailed Student’s t-test. A two-sided P value < 0.05 was considered statistically significant. All quantitative data are expressed as mean ± standard deviation (SD) from at least three independent biological replicates. Error bars in all graphs represent standard deviation (SD). The number of biological replicates (n) for each experiment is explicitly indicated in the corresponding figure legend. A *post-hoc* statistical power analysis for the CCK-8 proliferation assay was performed using G*Power (version 3.1). Based on the observed effect sizes and a sample size of n = 3 independent biological replicates with six technical replicates per group, the achieved statistical power (1 − β) exceeded 0.80 at a significance level of α = 0.05 for all primary comparisons, indicating adequate power to detect the reported differences.

## 3 Results

### 3.1 SOX10 distribution in four TNBC cell lines

**Figure 1:**
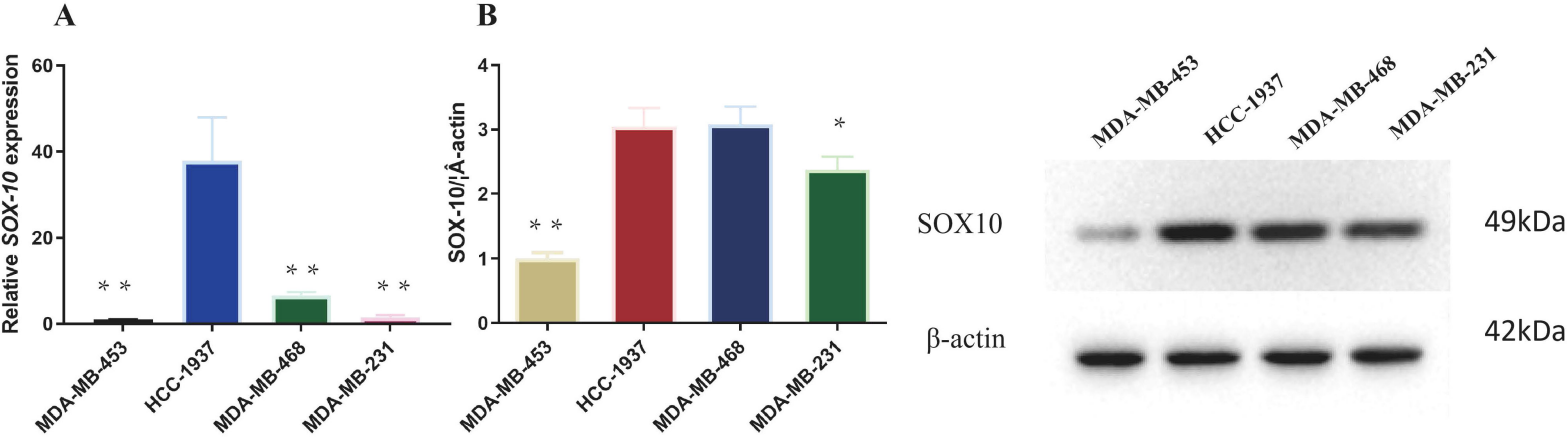
Endogenous expression of SOX10 in four TNBC cell lines. **(A)** Relative mRNA expression of SOX10 detected by qRT-PCR. **(B)** Protein expression of SOX10 detected by Western blot analysis. All experiments were independently repeated three times (n = 3 independent biological replicates), and representative results are shown. Statistical analysis was performed using one-way ANOVA with Tukey’s *post-hoc* test. *P < 0.05, **P < 0.01 vs. MDA-MB-453 cell line.

As shown in **Figures 1A, B**, SOX10 was predominantly expressed in HCC1937 cells and rarely detected in MDA-MB-453 cells. Consequently, HCC1937 cells (exhibiting the highest SOX10 expression) and MDA-MB-453 cells (exhibiting the lowest SOX10 expression) were selected for further experiments.

### 3.2 Effects of SOX10 knockdown and overexpression on the proliferation and metastasis of TNBC cells

**Figure 2:**
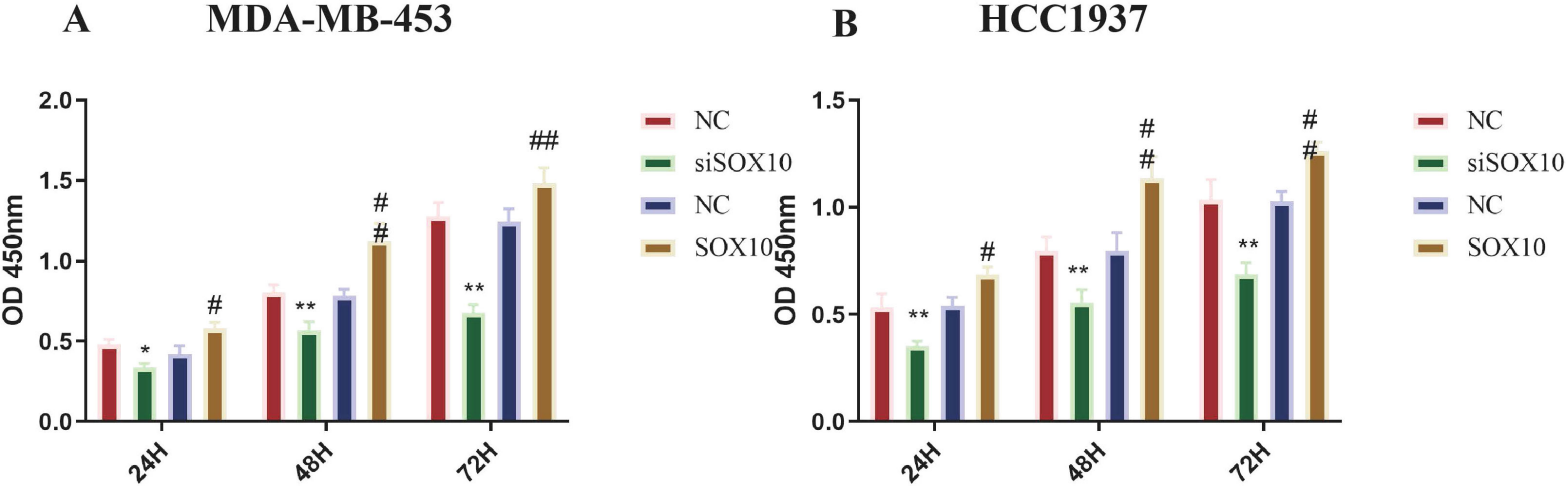
Effect of SOX10 knockdown and overexpression on the proliferation of TNBC cells. Cell proliferation was assessed at 24, 48, and 72 h post-transfection, with six technical replicate wells per group at each time point and three independent biological replicates (n = 3). Data are presented as mean ± SD. **(A)** Proliferation curve of MDA-MB-453 cells. **(B)** Proliferation curve of HCC1937 cells. Statistical analysis was performed using two-way ANOVA with Tukey’s *post-hoc* test for multiple comparisons at each time point. siNC vs. siSOX10, *P < 0.05, **P < 0.01; NC vs. SOX10-OE, ^#^*P* < 0.05, ^##^*P* < 0.01.

**Figure 3:**
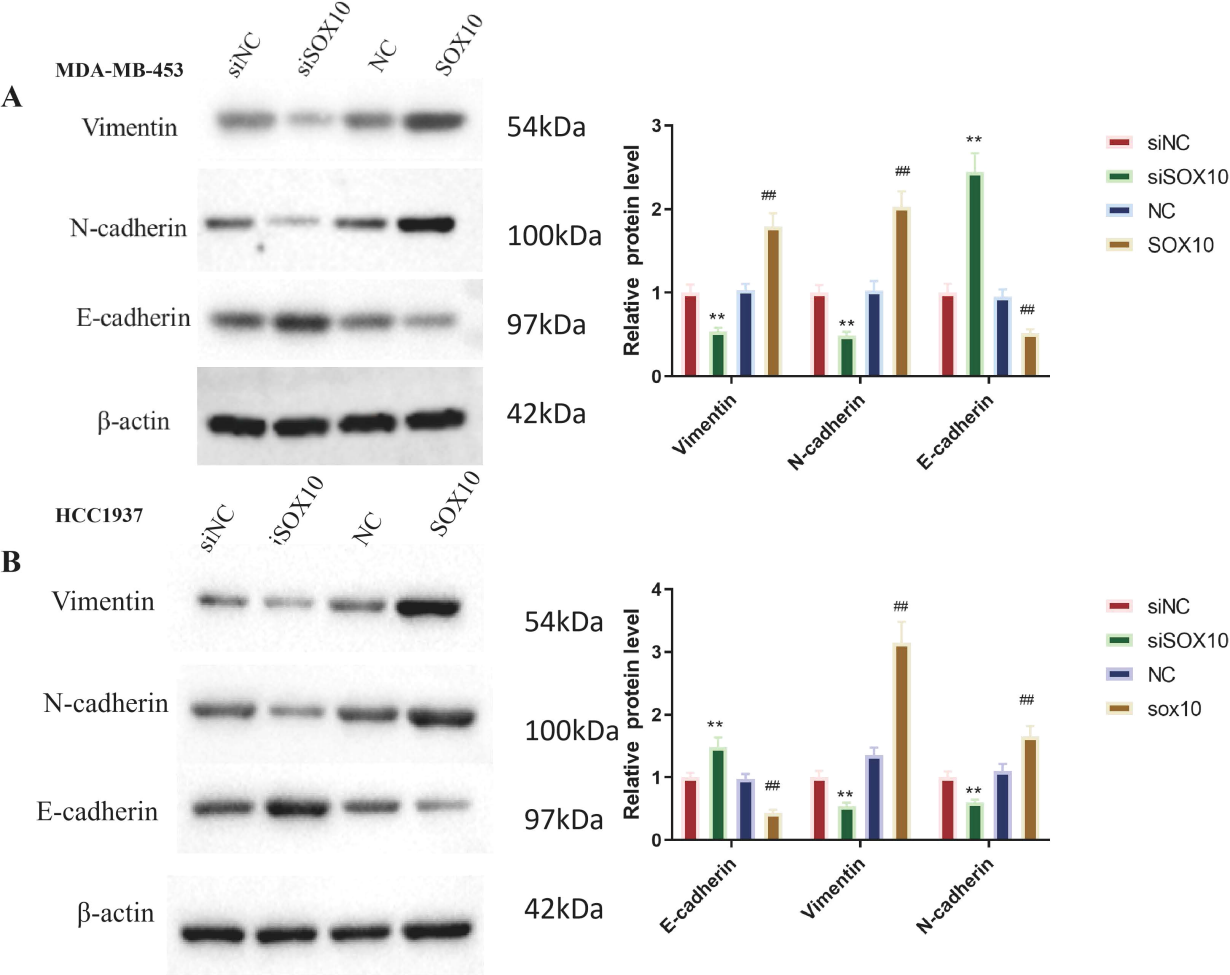
Effect of SOX10 knockdown and overexpression on the migration ability of TNBC cells. **(A)** Representative scratch images of TNBC cells at 0 and 24 h post-scratch (100× magnification), scale bar: 100 μm. **(B)** Quantification of cell migration rate, calculated from three random fields per well with three parallel measurements per field, and the experiment was independently repeated three times. Statistical analysis was performed using unpaired two-tailed Student’s t-test for two-group comparisons. siNC vs. siSOX10, **P < 0.01; NC vs. SOX10-OE, ^##^*P* < 0.01.

**Figure 4:**
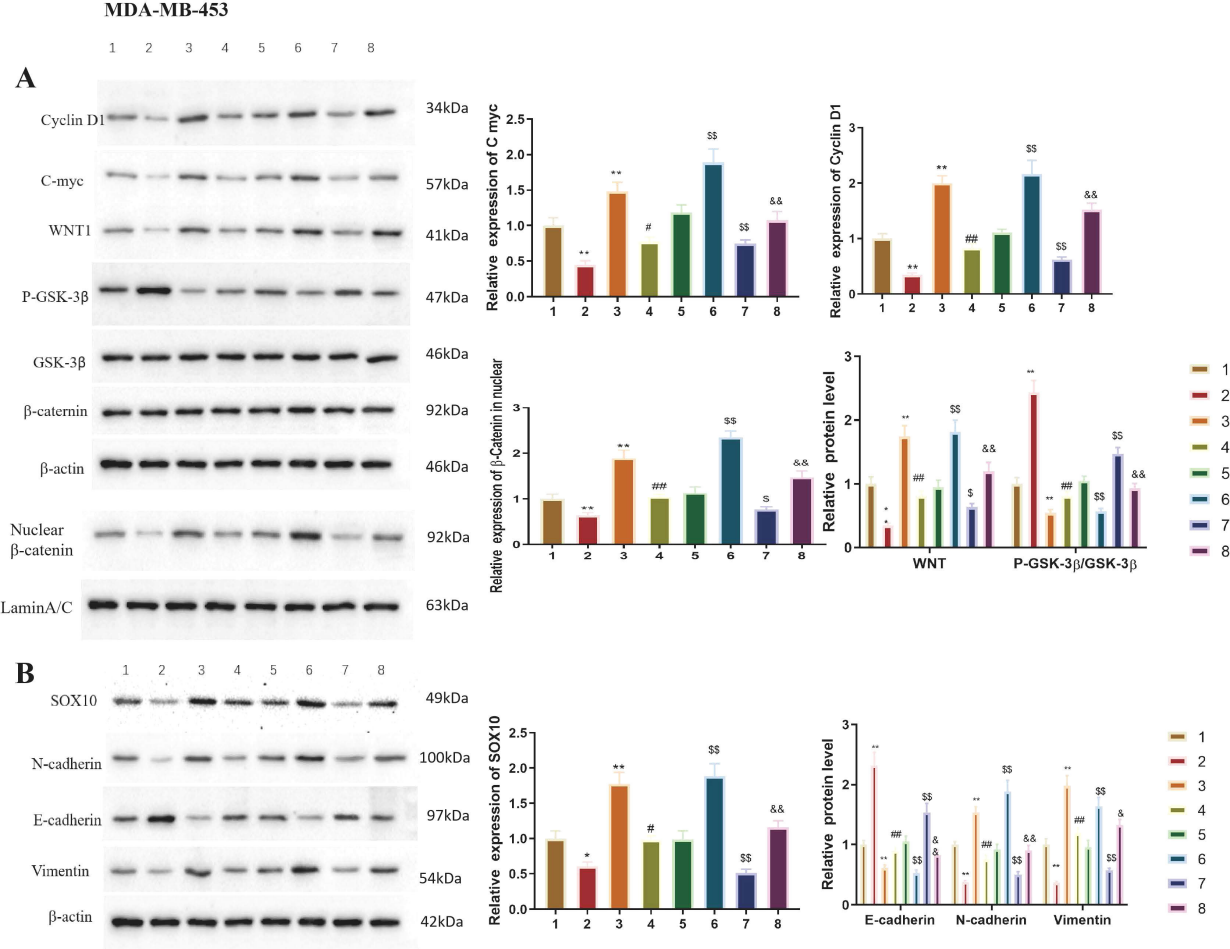
Effect of SOX10 knockdown and overexpression on the invasive ability of TNBC cells. **(A)** Representative images of crystal violet-stained invasive TNBC cells (200× magnification), scale bar: 100 μm. **(B)** Quantification of invasive cell number, with five non-overlapping random fields counted per chamber in a blinded manner, and the experiment was independently repeated three times. Statistical analysis was performed using unpaired two-tailed Student’s t-test for two-group comparisons. siNC vs. siSOX10, **P < 0.01; NC vs. SOX10-OE, ^##^*P* < 0.01, && indicates P<0.01; $$ indicates P<0.01 for statistical comparisons.

**Figures 2A, B** demonstrate that SOX10 knockdown significantly inhibited cellular growth at all time points (*p* < 0.05), while SOX10 overexpression significantly promoted cell proliferation (*p* < 0.05). The most significant difference was observed at 48 h, as cells entered the growth plateau stage thereafter. Additionally, we found that cells in the siSOX10 group showed a significantly decreased migration rate, whereas SOX10 overexpression notably accelerated cell migration (**Figures 3A, B**, *p* < 0.01). Transwell experiments revealed a significant decrease in cell invasion for both MDA-MB-453 and HCC1937 cells following siSOX10 transfection, while SOX10 overexpression led to an increase in cell invasion (**Figures 4A, B**, *p* < 0.05).

### 3.3 Effect of SOX10 on EMT-related and Wnt/β-catenin pathway-related protein expression in TNBC cells

**Figure 5:**
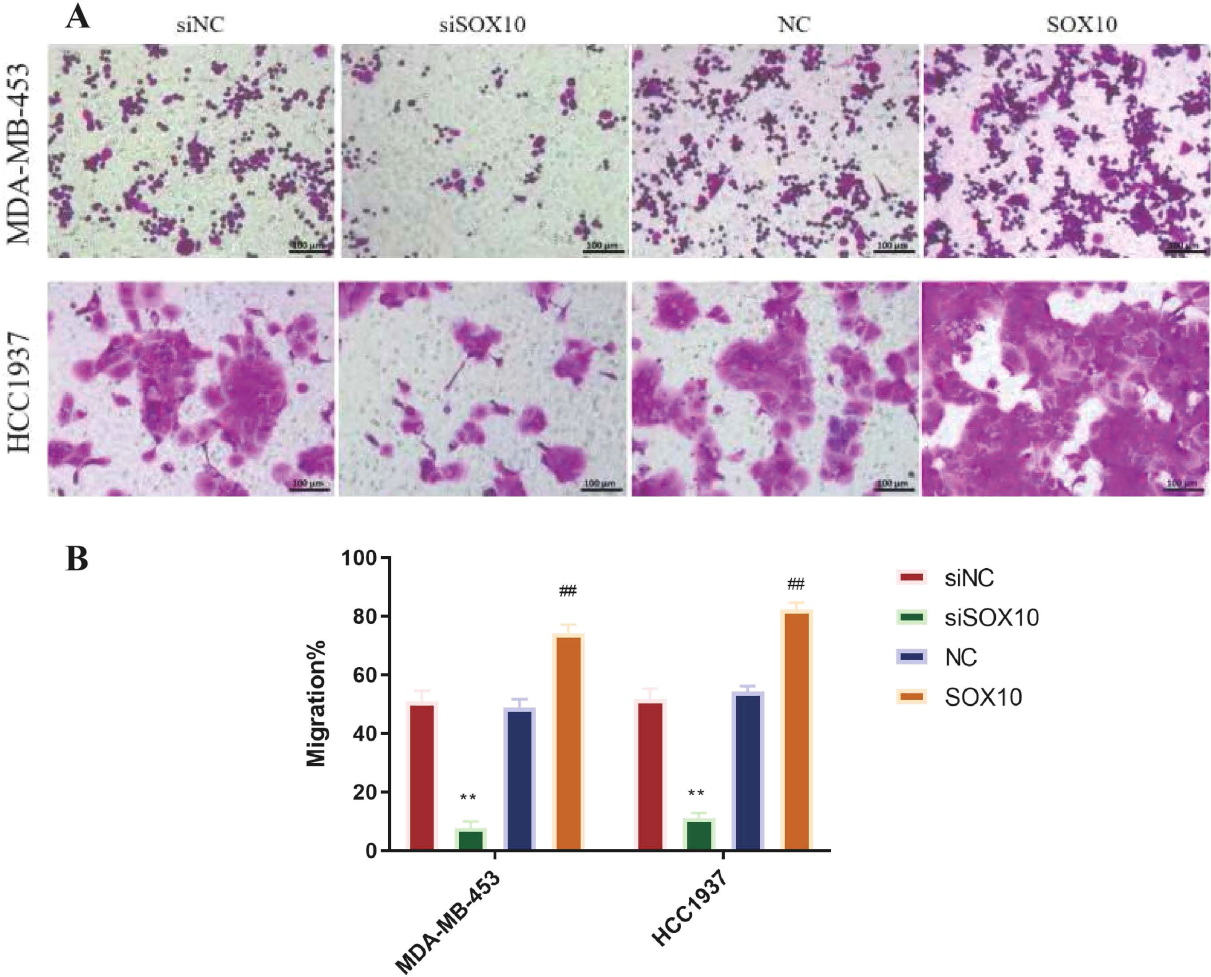
Effect of SOX10 knockdown and overexpression on EMT-related protein expression in TNBC cells. Protein expression of E-cadherin, N-cadherin, and Vimentin was detected by Western blot analysis, with band gray values normalized to the internal reference GAPDH. **(A)** Protein expression and quantification in MDA-MB-453 cells. **(B)** Protein expression and quantification in HCC1937 cells. All experiments were independently repeated three times (n = 3 independent biological replicates), and representative results are shown. Statistical analysis was performed using unpaired two-tailed Student’s t-test for two-group comparisons. siSOX10 vs. siNC, **P < 0.01; SOX10-OE vs. NC, ^##^*P* < 0.01.

EMT marker expression was analyzed in transfected MDA-MB-453 (**Figure 5A**) and HCC1937 (**Figure 5B**) cells. After overexpressing *SOX10*, Vimentin and N-cadherin protein levels significantly increased, while E-cadherin was notably downregulated (P<0.01). Conversely, knocking down *SOX10* evidently downregulated Vimentin and N-cadherin, but increased E-cadherin protein levels (P<0.01).

**Figure 6:**
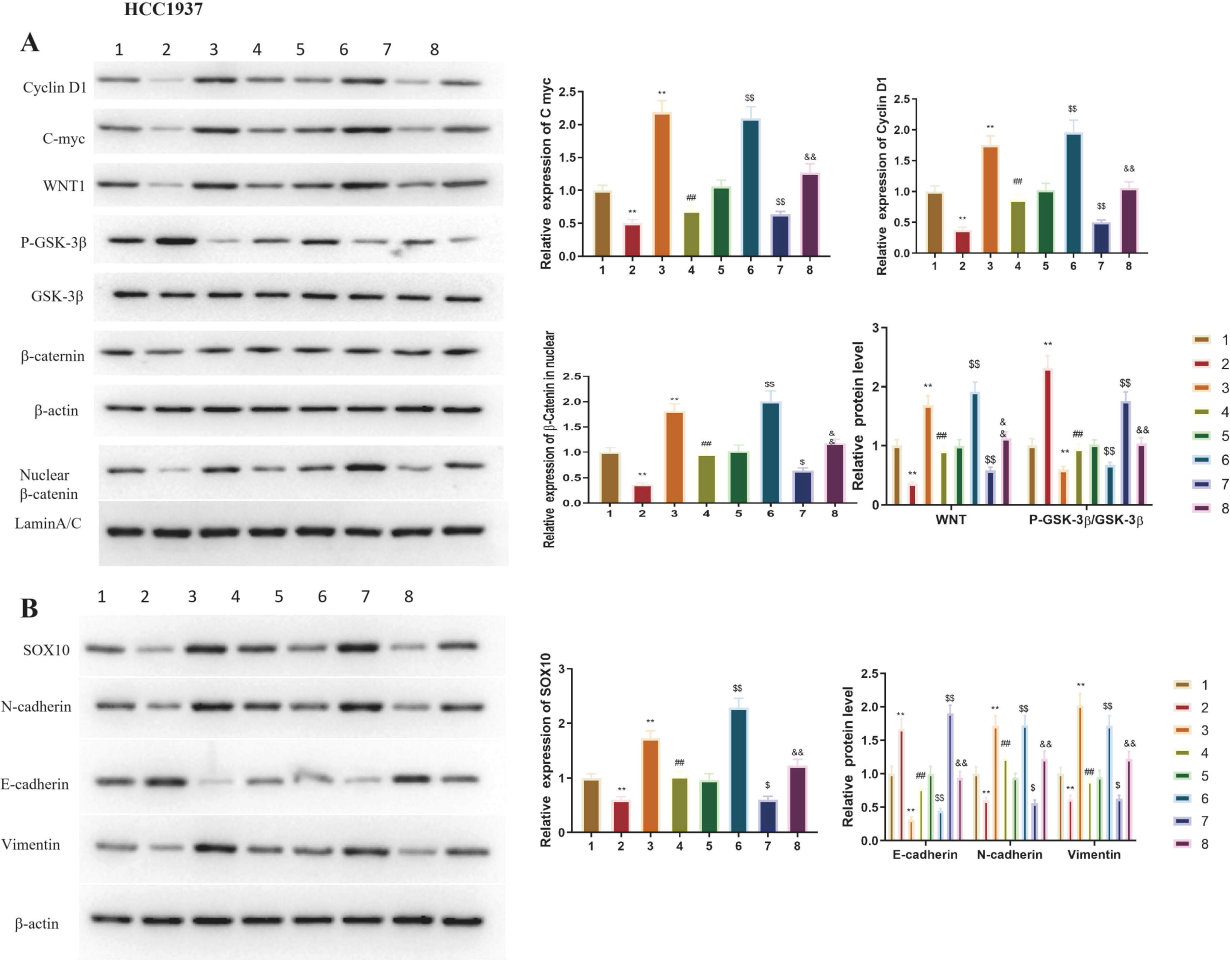
Effect of SOX10 knockdown and overexpression on classical Wnt/β-catenin pathway-related protein expression in TNBC cell lines. Protein expression of C-myc, CyclinD1, WNT1, phospho-GSK-3β (Ser9), and nuclear β-catenin was detected by Western blot analysis, with band gray values normalized to GAPDH (total protein) or Lamin B1 (nuclear protein, for β-catenin). **(A)** Protein expression and quantification in MDA-MB-453 cells. **(B)** Protein expression and quantification in HCC1937 cells. All experiments were independently repeated three times (n = 3 independent biological replicates), and representative results are shown. Statistical analysis was performed using unpaired two-tailed Student’s t-test for two-group comparisons. siSOX10 vs. siNC, **P < 0.01; SOX10-OEvs. NC, ^##^*P* < 0.01. && indicates P<0.01; $$ indicates P<0.01 for statistical comparisons.

In **Figures 6A, B**, overexpression of the *SOX10* gene notably induced C-myc, CyclinD1, and WNT1 proteins, with a significant increase in nuclear β-catenin protein, while P-GSK-3β protein expression decreased (P<0.01). The opposite effects were observed in cells with reduced *SOX10* gene expression (*p* < 0.01). The nuclear β-catenin/Lamin B1 ratio significantly increased with SOX10 overexpression and significantly decreased with SOX10 knockdown in both cell lines (P<0.01) (**Figures 6A, B**).

Consistent with the protein expression changes, qRT-PCR analysis revealed that SOX10 overexpression significantly upregulated the mRNA levels of WNT1 and β-catenin, while SOX10 knockdown significantly downregulated their mRNA levels in both MDA-MB-453 and HCC1937 cells (P<0.01). These results demonstrate that SOX10 regulates the Wnt/β-catenin pathway at both transcriptional and translational levels. These *in vitro* results indicate that SOX10 expression is associated with changes in EMT markers and Wnt/β-catenin pathway-related proteins in TNBC cells, although the underlying mechanistic basis remains to be established.

### 3.4 SOX10 activates the Wnt/β-catenin pathway to promote EMT in TNBC cells

**Figure 7:**
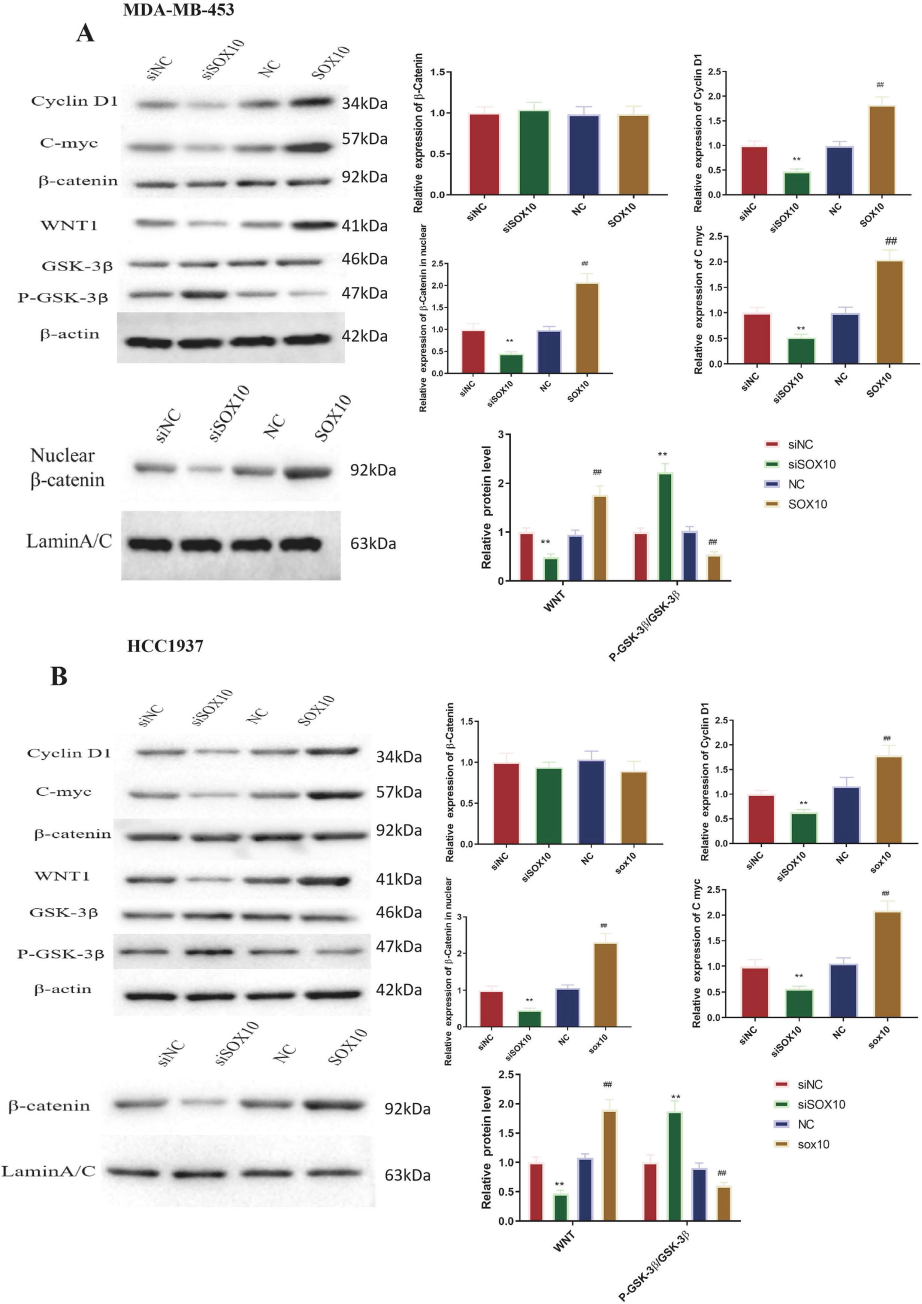
SOX10 promotes EMT in MDA-MB-453 cells via activating the classical Wnt/β-catenin signaling pathway. **(A)** Relative protein expression of Wnt/β-catenin pathway-related factors (WNT1, β-catenin, C-myc, and CyclinD1) in siSOX10 MDA-MB-453 cells treated with a Wnt activator, and SOX10-overexpressing MDA-MB-453 cells treated with a Wnt inhibitor. **(B)** Relative protein expression of EMT-related markers (E-cadherin, N-cadherin, and Vimentin) in the aforementioned treatment groups. All protein levels were detected by Western blot analysis, with band gray values normalized to GAPDH. All experiments were independently repeated three times (n = 3 independent biological replicates), and representative results are shown. Statistical analysis was performed using one-way ANOVA with Tukey’s *post-hoc* test for multiple comparisons among groups. Compared with MDA-MB-453+siNC, *P < 0.05, **P < 0.01; compared with MDA-MB-453+siSOX10, ^#^*P* < 0.05, ^##^*P* < 0.01; compared with MDA-MB-453+NC.

**Figure 8:**
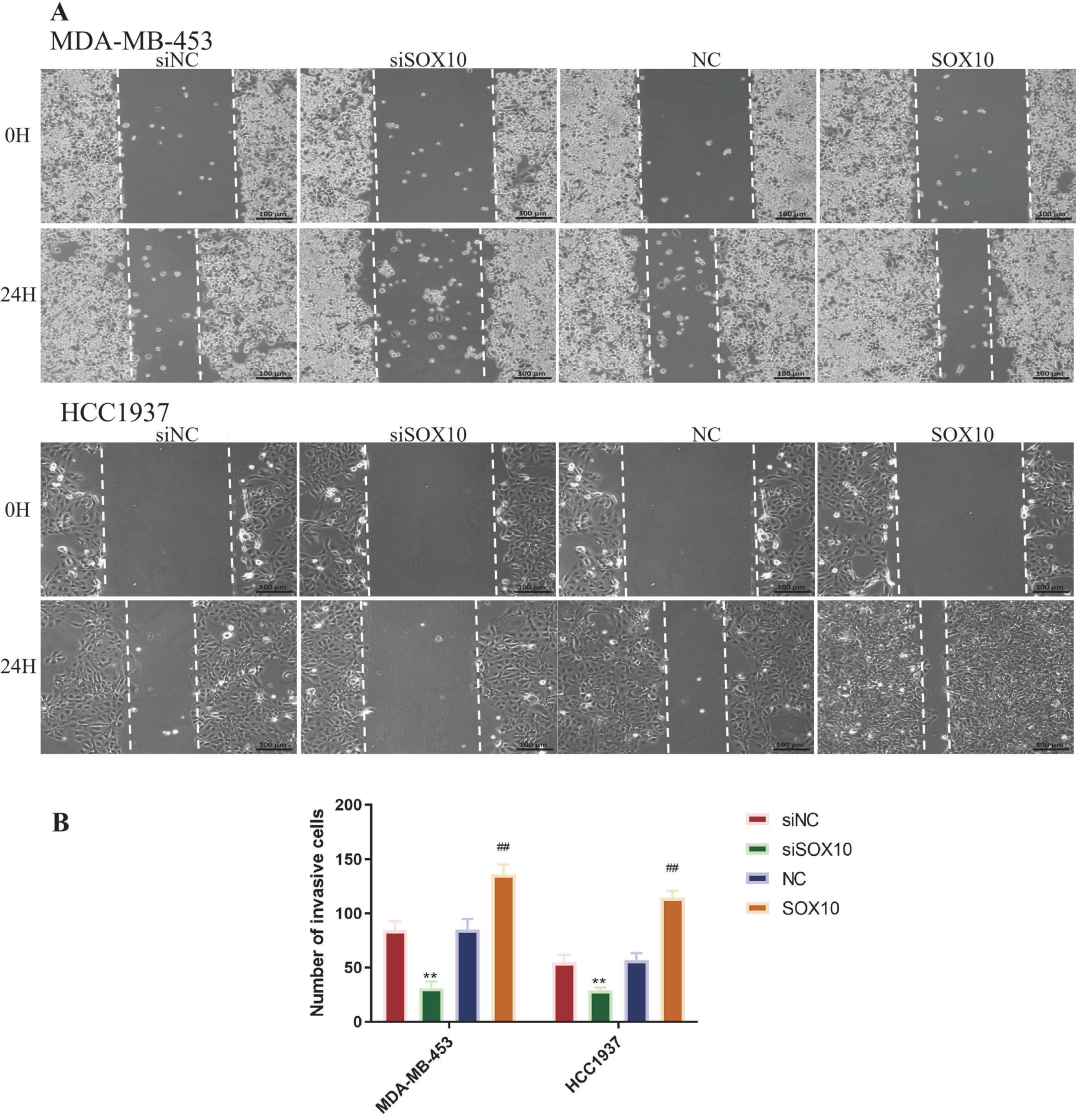
SOX10 promotes EMT in HCC1937 cells via activating the classical Wnt/β-catenin signaling pathway. **(A)** Relative protein expression of Wnt/β-catenin pathway-related factors (WNT1, β-catenin, C-myc, and CyclinD1) in siSOX10 HCC1937 cells treated with a Wnt activator, and SOX10-overexpressing HCC1937 cells treated with a Wnt inhibitor. **(B)** Relative protein expression of EMT-related markers (E-cadherin, N-cadherin, and Vimentin) in the aforementioned treatment groups. All protein levels were detected by Western blot analysis, with band gray values normalized to GAPDH. All experiments were independently repeated three times (n = 3 independent biological replicates), and representative results are shown. Statistical analysis was performed using one-way ANOVA with Tukey’s *post-hoc* test for multiple comparisons among groups. Compared with HCC1937+siNC, **P < 0.01; compared with HCC1937+siSOX10, ^##^*P* < 0.01; compared with HCC1937+NC.

The link between SOX10-induced EMT and the Wnt/β-catenin pathway was demonstrated in TNBC cells (**Figures 7A**, **8A**). Wnt activators were effective in siNC and siSOX10 groups, while Wnt inhibitors acted in overexpressed NC and overexpressed SOX10 groups (P<0.05). Moreover, in siSOX10-MDA-MB-453 and siSOX10-HCC1937 cells, Wnt activators significantly increased Vimentin and N-cadherin, while E-cadherin protein significantly decreased. Following inhibition of the Wnt/β-catenin pathway in SOX10-overexpressing MDA-MB-453 and HCC1937 cells, Vimentin and N-cadherin protein expression levels significantly decreased, and E-cadherin protein expression level significantly increased (**Figures 7B**, **8B**; P<0.05). Taken together, these results suggest that the effects of SOX10 on EMT-related markers are associated with Wnt/β-catenin pathway activity in TNBC cells, although the precise mechanistic relationship requires further investigation.

## 4 Discussion

Immunohistochemistry reveals SOX10 is only positively expressed in the myoepithelial cell nucleus of normal breast tissue, not in other breast cells (14). This is consistent with our preliminary findings that SOX10 is present only in myoepithelial cells surrounding lobules and ducts in normal breast tissue and is localized to the nucleus of these cells (15). Ali et al. (16) and Yoon et al. (17) found that SOX10 was predominantly positively expressed in basal BC and unclassified TNBC, with rare expression in other types of BC. We previously confirmed a significantly higher SOX10 positive rate in TNBC compared to other molecular subtypes of BC, at both cytological and histological levels (15). Many studies have confirmed that TNBC is a malignant tumor originating from breast myoepithelial cells (18, 19). Myoepithelial cells exist not only in mammary lobules and ducts but also in other organs. Studies have also found that many tumors derived from myoepithelium in these organs express SOX10 protein (20–22), which may explain the higher positive rate of SOX10 in TNBC.

SOX10 is involved in the occurrence and progression of multiple tumors, regulating cell proliferation, migration, invasion, and other biological behaviors (23). For instance, in nasopharyngeal carcinoma, SOX10 is significantly overexpressed in tumor tissues and cells; conversely, the absence of SOX10 significantly inhibits cell proliferation, metastasis, and EMT processes (6). In addition, SOX10 expression in bladder cancer tissues is significantly higher than in adjacent tissues, and it is associated with the clinicopathological characteristics of bladder cancer (clinical stage, histological grade, lymph node metastasis, etc.). SOX10 can also independently predict the overall survival prognosis and promote the proliferation, migration, and invasion of bladder cancer cells (23). The role of SOX10 in different tumors and its impact on cell biological behavior vary, and its mechanism of action is also not uniform.

As mentioned earlier, SOX10 acts as an oncogene in most tumors and is associated with a poor prognosis. In contrast, Kato et al. found that a low SOX10 level correlated with high venous infiltration in diffuse gastric cancer. Survival analysis showed that high venous infiltration is a statistically significant poor prognostic factor, indicating that a decrease in SOX10 can accelerate vascular invasion and lead to adverse outcomes in patients. Therefore, SOX10 may primarily function as a suppressor in such tumors (24). In breast tissue, the Notch signal transduction pathway is capable of maintaining stem cell characteristics and regulating cell differentiation and development. This pathway can promote the development of BC cells and drive BC progression. Inhibition of Notch receptors and ligands may become a new direction for BC treatment in the future (25–28). *In vitro* experiments using mouse mammary epithelial cells showed that the peroxisome-proliferator-activated receptor binding protein (*pBp*) gene is involved in mammary duct formation. Mutations in the *pBp* gene inhibited Notch4-mediated the growth of these cells, an effect that SOX10 expression could reverse. Moreover, the *pBp* gene was found to activate SOX10 to regulate Notch4-mediated growth and proliferation of mammary epithelial cells, indicating that SOX10 primarily controls Notch4-mediated proliferation in these cells (25). Although the SOX10-positive rate in TNBC is high, there is no evidence regarding the role of *SOX10* gene in primary TNBC expansion. Prognosis is related to tumor staging, lymph node metastasis and the number of metastases, vascular and perineural invasion, pathological grading, and tumor size. In our previous research involving 71 TNBC cases, we found that SOX10 positivity correlated with poor prognostic factors, such as tumor pathological grade, vascular and perineural invasion, Ki-67 proliferation index, clinical stage, and the number of lymph node metastases. Furthermore, patients in the SOX10-positive group showed a significantly reduced 5-year disease-free survival compared to the SOX10-negative group (15). Therefore, we speculated that SOX10 expression may be associated with a poor prognosis for TNBC and could become a new marker to predict TNBC prognosis and metastatic potential, potentially offering a target for TNBC treatment.

In this study, *in vitro* experiments showed that in both high-SOX10 (HCC1937) and low-SOX10 (MDA-MB-453) cell lines, SOX10 overexpression significantly enhanced cell proliferation, migration, and invasion, while SOX10 knockdown significantly reduced these abilities. This proved that the SOX10 gene promotes proliferation and metastasis of TNBC cells, as evidenced by both overexpression and knockdown experiments. Based on these results, we further verified that *SOX10* overexpression enhanced the malignant potential of TNBC cells at both the histological and *in vitro* cytological levels.

Following EMT in tumor cells, epithelial cells exhibit relatively strong adhesion, whereas cells with a stromal phenotype show significantly reduced adhesion. Consequently, tumor cells undergoing EMT not only change their morphology and structure, but also exhibit significant changes in adhesion, migration, and invasion abilities, thereby enhancing their ability to infiltrate and migrate (7). The SOX10 gene promotes tumor progression and metastasis by inducing EMT in nasopharyngeal carcinoma cells (6). In our previous research, we found that SOX10-positive TNBC cells have high expression of Vimentin and N-cadherin. In this experiment, we found that SOX10 can promote EMT in TNBC cells, leading to the speculation that the SOX10 gene may also promote tumor progression and metastasis by inducing EMT. It is important to acknowledge that the EMT characterization in the present study was based on a limited panel of protein markers (E-cadherin, N-cadherin, and Vimentin), which reflect protein-level changes indicative of an EMT-like phenotypic shift but do not constitute a comprehensive assessment of EMT status. A thorough characterization would ideally include analysis of key EMT transcription factors (e.g., Snail, Slug, Twist, ZEB1), functional assays such as morphological assessment, and ideally single-cell or transcriptomic approaches to capture EMT heterogeneity. Therefore, the changes in EMT-associated markers observed in the present study should be interpreted as indicative of EMT-like alterations rather than definitive EMT induction.

The name Wnt was derived from a combination of the *Drosophila wingless* (*wg*) gene and the nude mouse *Int-1* gene. The abnormal signal transduction function of Wnt pathway can lead to developmental disorders and numerous diseases. Among the three types of Wnt pathways (29), abnormalities in the classical Wnt/β-catenin pathway are most common in human malignant tumors, critically promoting the proliferation, differentiation, and metastasis of cancer cells. As the main effector of the classical Wnt pathway, *β-catenin* is abnormally expressed in many tumors (30–32). When the Wnt/β-catenin pathway is inactive, β-catenin is mainly localized on the cell membrane and regulates cell-to-cell adhesion by binding to the intracellular peptide segment of E-cadherin. In the cytoplasm, a small amount of free β-catenin typically forms complexes with GSK3, Axin, APC, and DSH, and is phosphorylated and degraded, resulting in low cytoplasmic levels. However, when cells receive paracrine or autocrine Wnt signals, Wnt proteins bind to the transmembrane receptors Frz and LRP5/6 on the cell membrane, thereby activating the intracellular signaling pathway, leading to the inactivation of the complex composed of GSK3, Axin, APC, and DSH, preventing the degradation of the free β-catenin located in the cytoplasm. When β-catenin gradually accumulates to a certain amount, it crosses the membrane and enters the nucleus, leading to abnormal nuclear β-catenin accumulation. This nuclear β-catenin can then bind to T-cell factor/lymphoenhancer binding factor (TCF/LEF) transcription factors, thereby activating a series of Wnt target genes (C-myc, CyclinD1, WNT1, P-GSK-3β, etc.). These classic and important molecular changes typically occur when the classical Wnt/β-catenin signaling pathway is activated (33).

Many factors are associated with the Wnt pathway, and some Wnt proteins, such as Wnt1 and Wnt5A, transduce signals via non-classical Wnt pathways. Research has found that non-classical Wnt pathways can also induce EMT in some tumor cells. Studies of EMT in malignant melanoma cells have found that Wnt5A can promote the expression of Snail and vimentin via the protein kinase C (PKC) pathway, downregulate E-cadherin, and induce EMT in these cells (34).

Several limitations should be acknowledged. First, this study is restricted to *in vitro* cell models, and the observed functional effects have not been validated *in vivo*. Future studies will employ xenograft mouse models to assess the impact of SOX10 manipulation on tumor growth, invasion, and metastasis *in vivo*. Second, the clinical relevance of the SOX10–Wnt/β-catenin axis has not been confirmed in patient samples; future work will examine the correlation between SOX10 expression and nuclear β-catenin accumulation in TNBC patient specimens, as well as perform survival and pathway correlation analyses using publicly available TNBC cohorts such as TCGA and GEO datasets. These future studies will be essential to establish the translational significance of our current findings.

Although SOX10 has been reported to promote EMT and activate Wnt/β-catenin signaling in other cancers, such as nasopharyngeal carcinoma and hepatocellular carcinoma, its functional role and mechanistic involvement in TNBC remain poorly defined. Our study provides the first evidence that SOX10 promotes EMT and malignant behaviors in TNBC cells through upstream activation of the Wnt/β-catenin pathway, as demonstrated by rescue experiments using Wnt activators and inhibitors. In this study, we found that overexpression of *SOX10* in TNBC cells significantly upregulated nuclear β-catenin protein, along with other Wnt/β-catenin-targeted proteins such as C-myc, CyclinD1, WNT1, and P-GSK-3β. Conversely, knockdown of *SOX10* resulted in the significant downregulation of these proteins. Therefore, *SOX10* is associated with activation of the classic Wnt/β-catenin signaling pathway in TNBC cells, potentially through transcriptional upregulation of WNT1 and its downstream targets, although direct regulatory mechanisms remain to be confirmed. While our results show that SOX10 upregulates WNT1 and other Wnt/β-catenin pathway components at the transcriptional level, whether this regulation is direct or mediated through intermediate factors remains to be determined. Future studies employing ChIP-seq or luciferase reporter assays will be essential to determine whether SOX10 directly binds to the promoters of WNT1 or other Wnt pathway genes, thereby confirming direct transcriptional regulation. The absence of ChIP or luciferase reporter assays in this study limits our ability to confirm direct promoter binding or transcriptional activation by SOX10. It is important to note that this study is limited to *in vitro* models and does not include *in vivo* validation. The functional effects of SOX10 on tumor growth, invasion, and metastasis observed in cell lines have not been confirmed in animal models such as xenograft or orthotopic systems. Therefore, while our data strongly suggest that SOX10 promotes malignant behaviors through Wnt/β-catenin activation, these findings require further validation *in vivo* to establish their physiological and pathological relevance. Future studies will focus on establishing xenograft models to assess tumor growth and metastatic potential in response to SOX10 modulation. A notable limitation of this study is that functional experiments were conducted in only two TNBC cell lines. Although the four cell lines (HCC1937, MDA-MB-231, MDA-MB-468, and MDA-MB-453) were systematically screened for SOX10 expression and the two lines with the most contrasting expression levels were selected to maximize the dynamic range of the functional analysis, the findings may not fully capture the biological diversity of TNBC. As TNBC is a highly heterogeneous disease encompassing multiple molecular subtypes with distinct gene expression profiles and signaling pathway dependencies (35), the generalizability of our conclusions may be limited. HCC1937 (basal-like 1 subtype) and MDA-MB-453 (luminal androgen receptor subtype) represent different molecular subtypes (36), partially addressing TNBC heterogeneity; however, validation in additional TNBC lines such as MDA-MB-231 and MDA-MB-468, as well as comparison with non-TNBC breast cancer cell lines, will be necessary in future studies to more definitively establish the role of the SOX10–Wnt/β-catenin axis across the full spectrum of TNBC. Furthermore, proliferation inhibitors such as mitomycin C were not applied prior to the scratch assay; therefore, the possibility that cell proliferation partially contributed to wound closure cannot be entirely excluded (37). Specifically, the absence of mitomycin C or other anti-proliferative pre-treatment means that the contribution of cell proliferation to wound closure cannot be definitively excluded, a standard practice in studies where pure migration is the primary endpoint. While the 24-h observation window and corroborating Transwell migration data offer partial mitigation, future studies employing proliferation-inhibited scratch assays or real-time live-cell imaging would more rigorously isolate the migratory component. The current scratch assay results should therefore be interpreted as reflecting overall wound closure capacity, which includes both migratory and potentially proliferative contributions. Nevertheless, the relatively short 24-h observation window limits the extent to which proliferation could confound the migration measurements (38), and the consistent results obtained from the Transwell migration assay, which is independent of proliferative activity, provide additional support for the conclusion that SOX10 promotes TNBC cell migration. Currently, research on the mechanism of *SOX10*-induced EMT in tumor cells is limited. The only existing study reported that *SOX10* can induce EMT in nasopharyngeal carcinoma, but its mechanism was not further explored. In our research, proteins involved in EMT were downregulated in TNBC cells with SOX10 overexpression and Wnt/β-catenin suppression. Conversely, these proteins were upregulated in TNBC cells with SOX10 knockdown and Wnt/β-catenin activation. Therefore, we speculate that SOX10 in TNBC induces EMT in tumor cells through the classic Wnt/β-catenin pathway, thereby accelerating tumor invasion and metastasis. Importantly, our rescue experiments demonstrate that the effects of SOX10 on EMT are dependent on Wnt/β-catenin signaling. Activation of the pathway reversed the EMT suppression caused by SOX10 knockdown, while inhibition of the pathway attenuated the EMT induction by SOX10 overexpression. These observations are consistent with a functional link between SOX10 and Wnt/β-catenin-mediated EMT regulation, although definitive causal evidence would require additional mechanistic studies such as ChIP assays or transcriptional reporter experiments.

## Data Availability

The raw data supporting the conclusions of this article will be made available by the authors, without undue reservation.
